# Supplementary material for: Structural insights into the Venus flytrap mechanosensitive ion channel Flycatcher1
Source: Nat Commun. 2022 Feb 14;13:850. doi: 10.1038/s41467-022-28511-5 (PMC8844309; doi:10.1038/s41467-022-28511-5)
Supplement: Supplementary file 1 — Supplementary Information [file 41467_2022_28511_MOESM1_ESM.pdf]

Supplementary Information

Structural Insights into the Venus flytrap Mechanosensitive Ion Channel Flycatcher1

Sebastian Jojoa-Cruz, Kei Saotome, Che Chun (Alex) Tsui, Wen-Hsin Lee, Mark S. P. Sansom, Swetha E. Murthy, Ardem Patapoutian, Andrew B. Ward

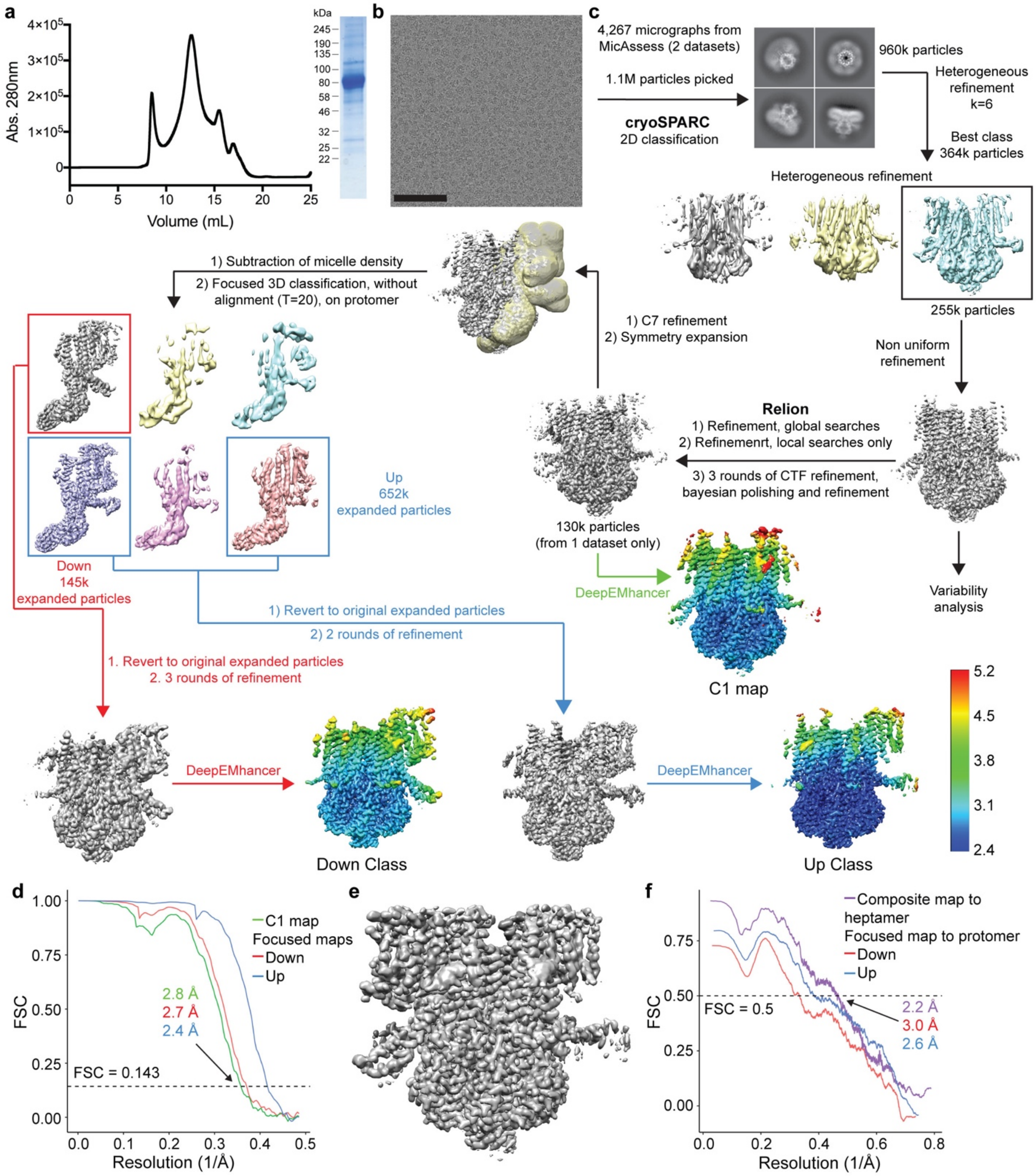

Supplementary Figure 1: Purification and cryo-EM data processing of FLYC1.
a, Size exclusion chromatography trace (left) and SDS-PAGE (right, uncropped gel in Source Data) from FLYC1 purification. Molecular weight of FLYC1 protomer is approximately 86kDa. **b,** Representative cryo-EM micrograph. Black bar is 100nm. Micrographs from two separate purifications and image collections were pooled together for cryo-EM analysis. **c,** Cryo-EM processing workflow. **d,** FSC plot of C1 and focused maps. **e,** Final composite map obtained by using the C1 and focused maps. **f,** Map to model FSC plot of composite and focused maps. Only the protomer was used for the calculation for the focused maps.

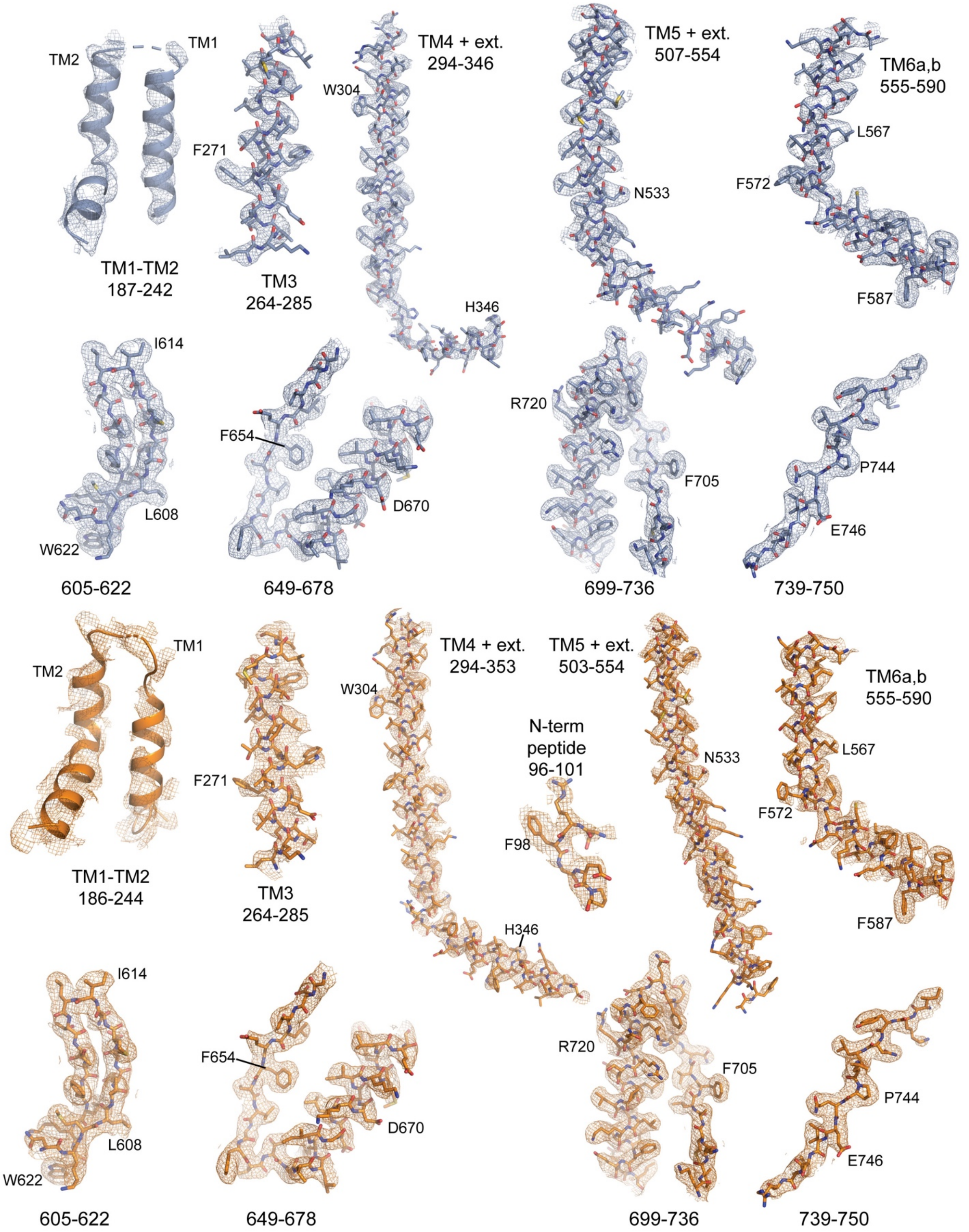

Supplementary Figure 2: Fit of FLYC1 model to map.
Fit of FLYC1 model for the up (top, blue) and down (bottom, orange) classes to composite map. Map was contoured at a threshold of 1 (for N-term peptide in down class) or 4 σ.

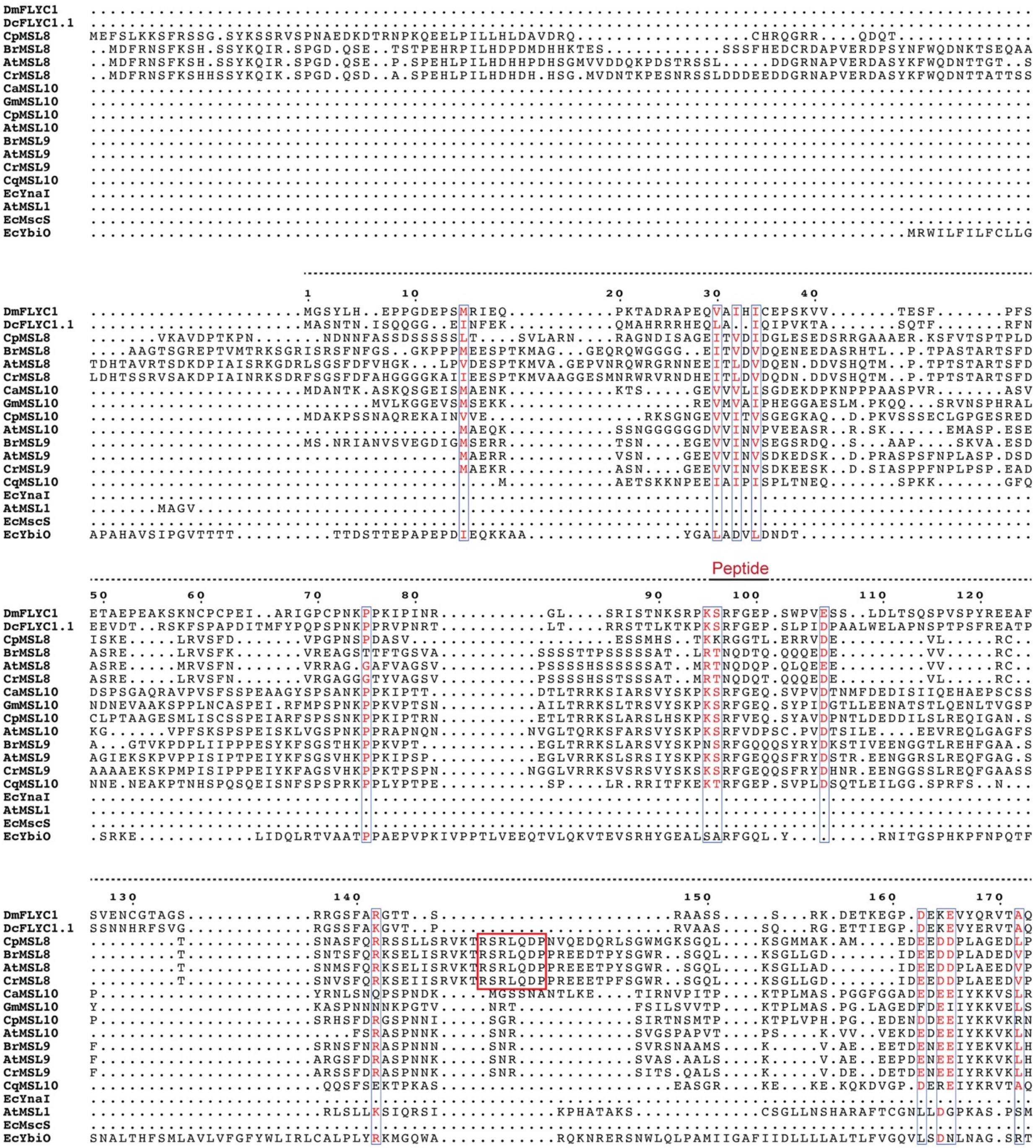
Peptide
DmFLYC1
DcFLYC1.1
CpMSL8
BrMSL8
AtMSL8
CrMSL8
CaMSL10
GmMSL10
CpMSL10
AtMSL10
BrMSL9
AtMSL9
CrMSL9
CqMSL10
EcYnaI
AtMSL1
EcMscS
EcYbiO

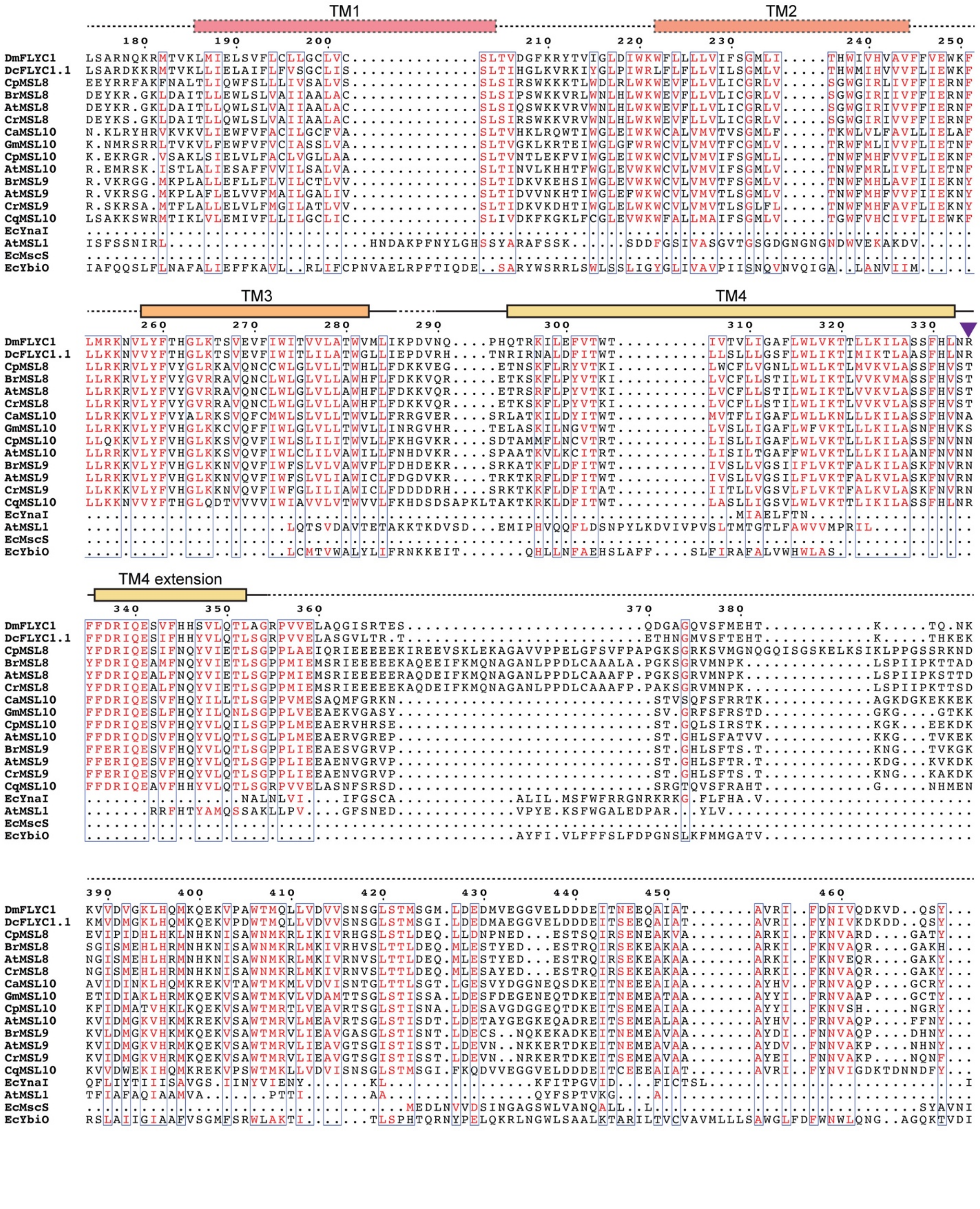
TM1
TM2
TM3
TM4
TM4 extension
DmFLYC1
DcFLYC1.1
CpMSL8
BrMSL8
AtMSL8
CrMSL8
CaMSL10
GmMSL10
CpMSL10
AtMSL10
BrMSL9
AtMSL9
CrMSL9
CqMSL10
EcYnaI
AtMSL1
EcMscS
EcYbiO

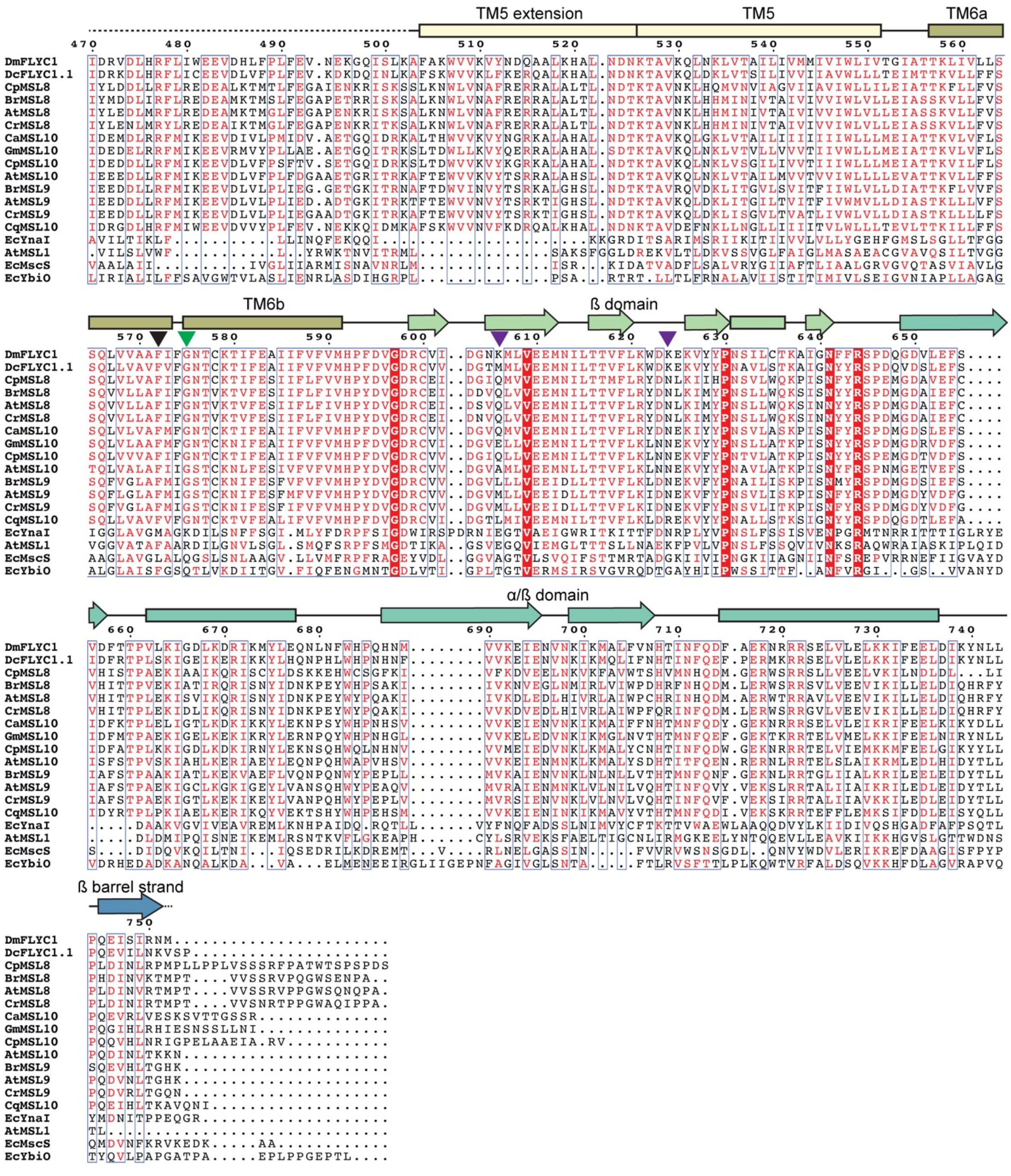

Supplementary Figure 3. Protein sequence alignment of (Dm)FLYC1.

Sequence alignment of DmFLYC1 (GenBank ID: QNN26181), *Drosera capensis* FLYC1.1 (QNN26182), *Carica papaya* (Cp)MSL8 (XP_021903761), *Brassica rapa* (Br)MSL8 (XP_018511053), AtMSL8 (NP_001318236), *Capsella rubella* (Cr)MSL8 isoform X1 (XP_023642637), *Coffea arabica* (Ca)MSL10-like (XP_027081862), *Glycine max* (Gm)MSL10 (XP_006578090), CpMSL10 (XP_021890723), AtMSL10 (NP_001119212), BrMSL9 (XP_009131740), AtMSL9 (NP_001331595), CrMSL9 (XP_006287126), *Chenopodium quinoa*

(Cq)MSL10-like (XP_021738929), EcYnaI (WP_000559900), AtMSL1 (NP_567165), EcMscS (WP_000389818), EcYbiO (WP_001267253). Secondary structure of FLYC1 represented at the top of each block, colored accordingly to Figure 1d. Dashed lines represent areas not built in the model. TM1 and TM2 are assigned based on TOPCONS predictions. TM4 and TM5 extensions form the TM4-TM5 linker. Arrow heads point to residue lining the smaller pore constriction (black), glycine hinge (green) or residues mutated in this study (purple). Red box surrounds residues of MSL8 homologs that align to the N-terminus peptide in alignment (not shown) that does not include AtMSL1 or bacterial homologs.

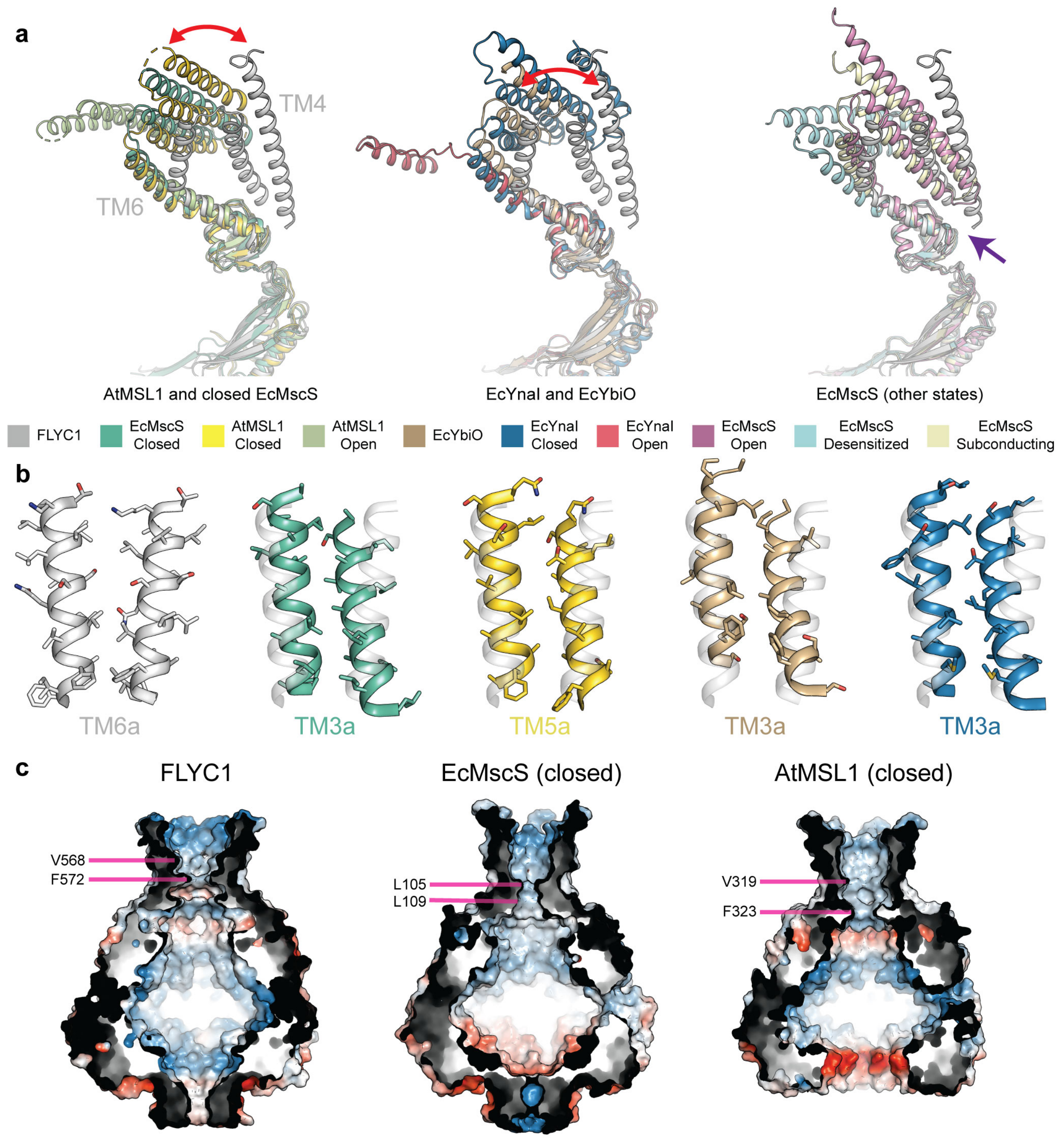

Supplementary Figure 4. Comparison of FLYC1 with solved homologs.
a, Superposition of FLYC1 subunit and reported structures of homologs (aligned on cytoplasmic domain). Red arrow show difference in rotation between FLYC1 and other homologs. Purple arrow points to the interaction between TM4-5, TM1-2 in EcMscS, and the pore helix for FLYC1 and EcMscS in both open and subconducting states. TM1-TM3 of FLYC1 are not shown. **b,** Alignment based on TM6a of two adjacent pore helices of FLYC1 and other homologs showing the presence of bulkier sidechains. PDB IDs used are: EcMscS closed state (2OAU), AtMSL1 closed state (6VXM), AtMSL1 open state (6VXN), EcYbiO (7A46), EcYnaI closed state (6ZYD), EcYnaI open state (6ZYE), EcMscS open state (5AJI), EcMscS desensitized state (6VYM), and EcMscS subconducting state (6VYL). **c,** Vertical section of surface representation

of cytoplasmic cage and pore helix colored by electrostatic potential for FLYC1 (left), closed EcMscS (middle) and closed AtMSL1 (right). Residues that form the hydrophobic gate in EcMscS and the corresponding residues in FLYC1 and AtMSL1 are labeled.

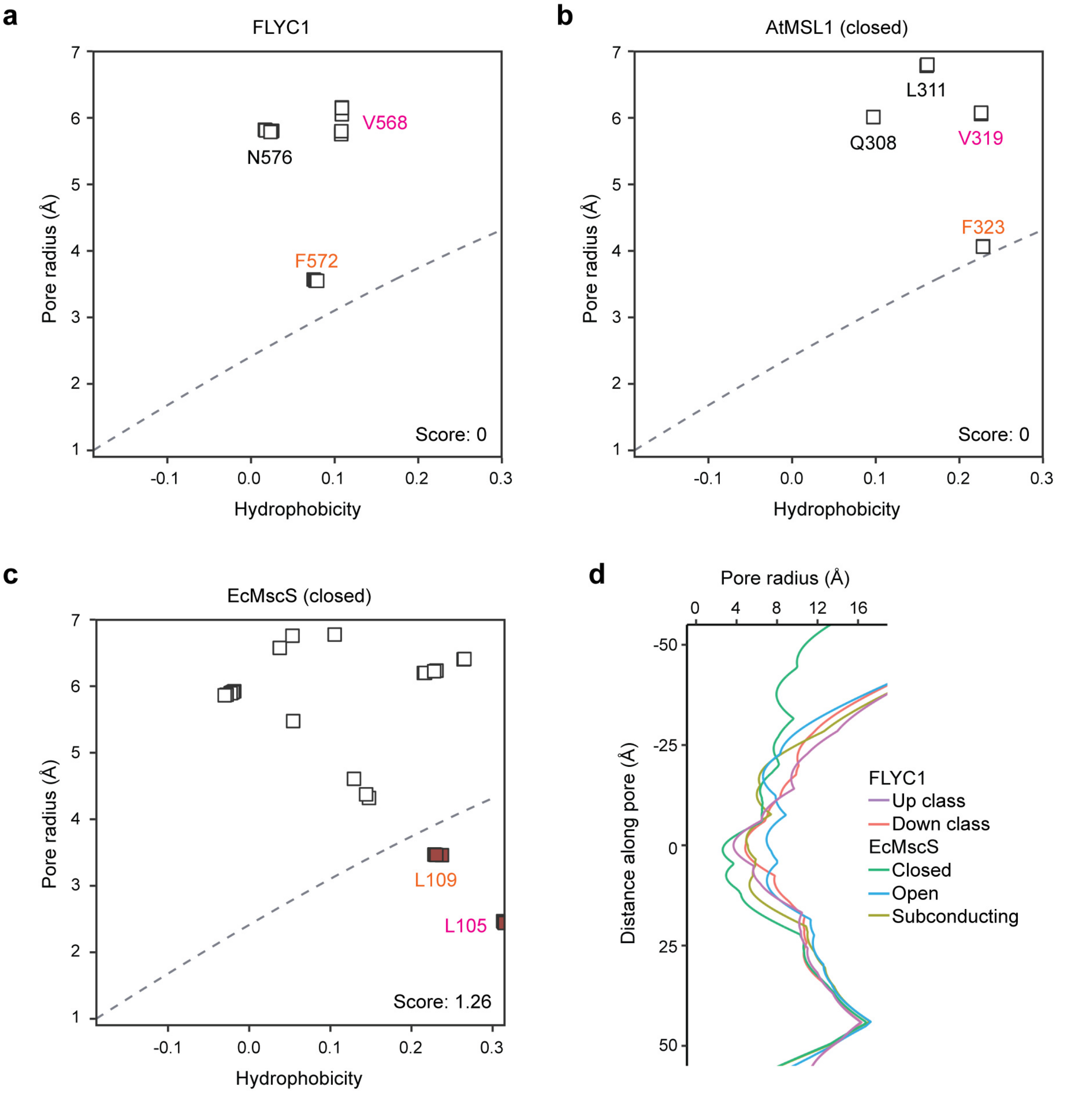

Supplementary Figure 5. Comparison of FLYC1 and homologs pore profile.
Heuristic prediction of likelihood of pore wetting of FLYC1 (**a**), closed AtMSL1 (**b**) and closed EcMscS (**c**). A score greater than 0.55 predicts the presence of at least one energetic barrier to water permeation in the channel pore. Residues that form the hydrophobic gate in EcMscS are labeled in pink and orange. The corresponding residues in FLYC1 and AtMSL1 follow the same coloring. **d,** Pore profile of hypothetical C7 FLYC1 conformations in comparison to EcMscS states.

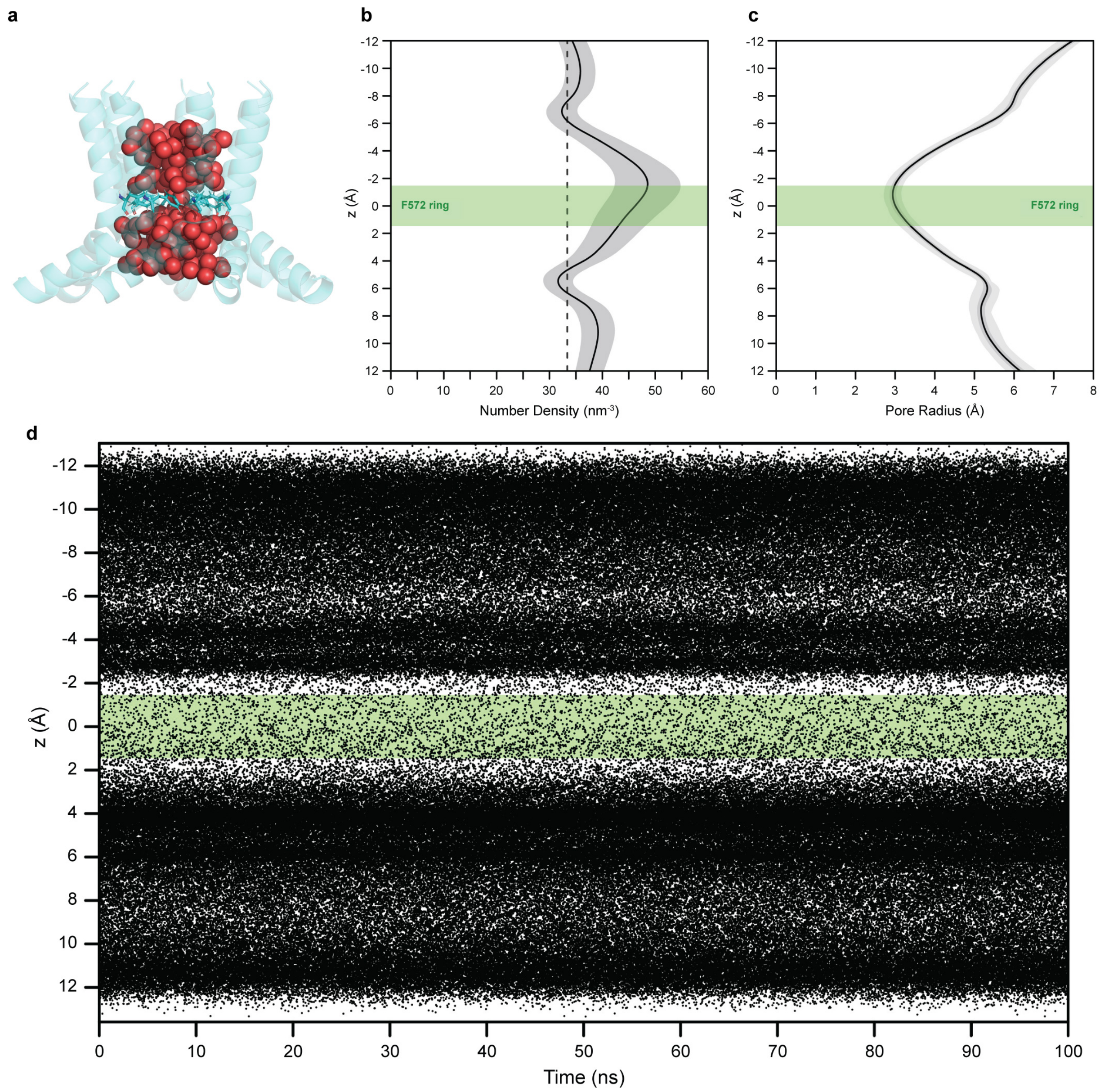

Supplementary Figure 6. The channel pore has no tendency to dewet in simulations (in the absence of an applied electric field), in accordance with our heuristic predictions.
a, A representative snapshot of the pore with water oxygen atoms represented by red spheres. The pore lining TM6 helices (pale cyan) and the ring of F572 residues are shown. **b,** Water number density (shown on the horizontal axis in units of waters nm^{-3}, with the curve corresponding to the mean ± standard deviation from a 100 ns simulation) along the pore axis (vertical) with the region around the ring of F572 residues (centred at $z = 0$) indicated in green. **C,** The corresponding pore radius profile. The analyses in **b** and **c** were derived from the simulation data using the CHAP (Channel Annotation Package) software (described in detail at https://www.channotation.org/). **d,** Position of water oxygen along the *z*-axis in the pathway throughout a 100-ns simulation (2501 frames).

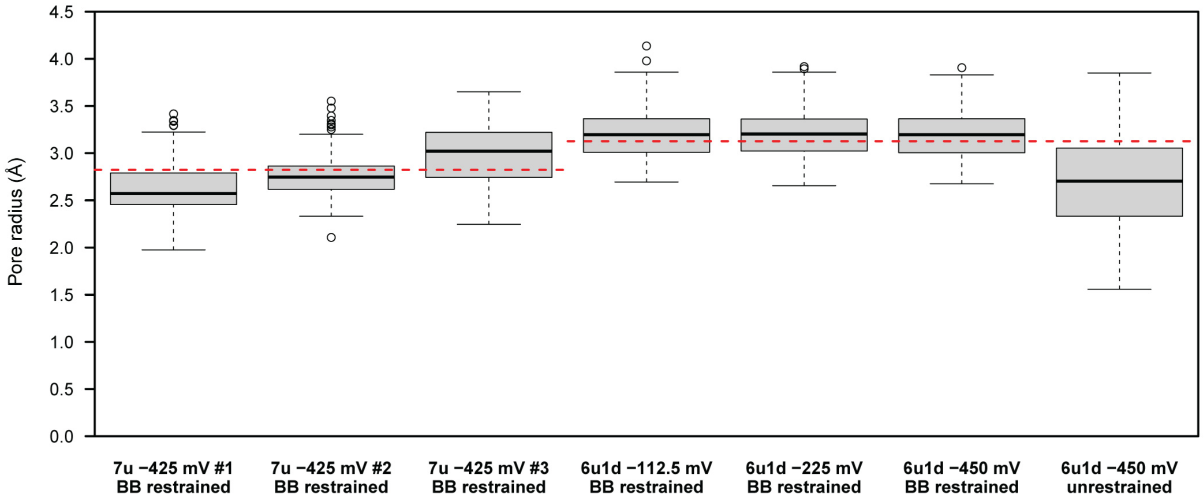

Supplementary Figure 7. The pore constriction geometry measured with the CHAP package for simulations using the 'all-up' (7u) and composite '6 up 1 down' (6u1d) model as the starting structure.

Boxplots of the minimum pore radius of the central ion-conducting pathway with different simulation setups. Each boxplot represents an individual 100-ns simulation (n = 1 for each stated condition, the three simulations with –425 mV potential difference of the 'all-up' model are analyzed and plotted independently), where frames from the last 90 ns are sampled. The box length represents the interquartile range of the frames sampled (226 frames) while the whiskers cover 1.5 times of the interquartile range from the box. Data outside this range are plotted as points. The center line of the box is the median. Simulations starting with the '6 up 1 down' model resulted in a more consistent pore constriction geometry (red dashed line indicates the starting value, at 3.1 Å), compared to the 'all-up' model (starting value 2.8 Å). The fully unrestrained simulation resulted in an overall narrowing of the constriction, accompanied by the loss of local symmetry and a slight compression into an elliptical shape. BB: backbone.

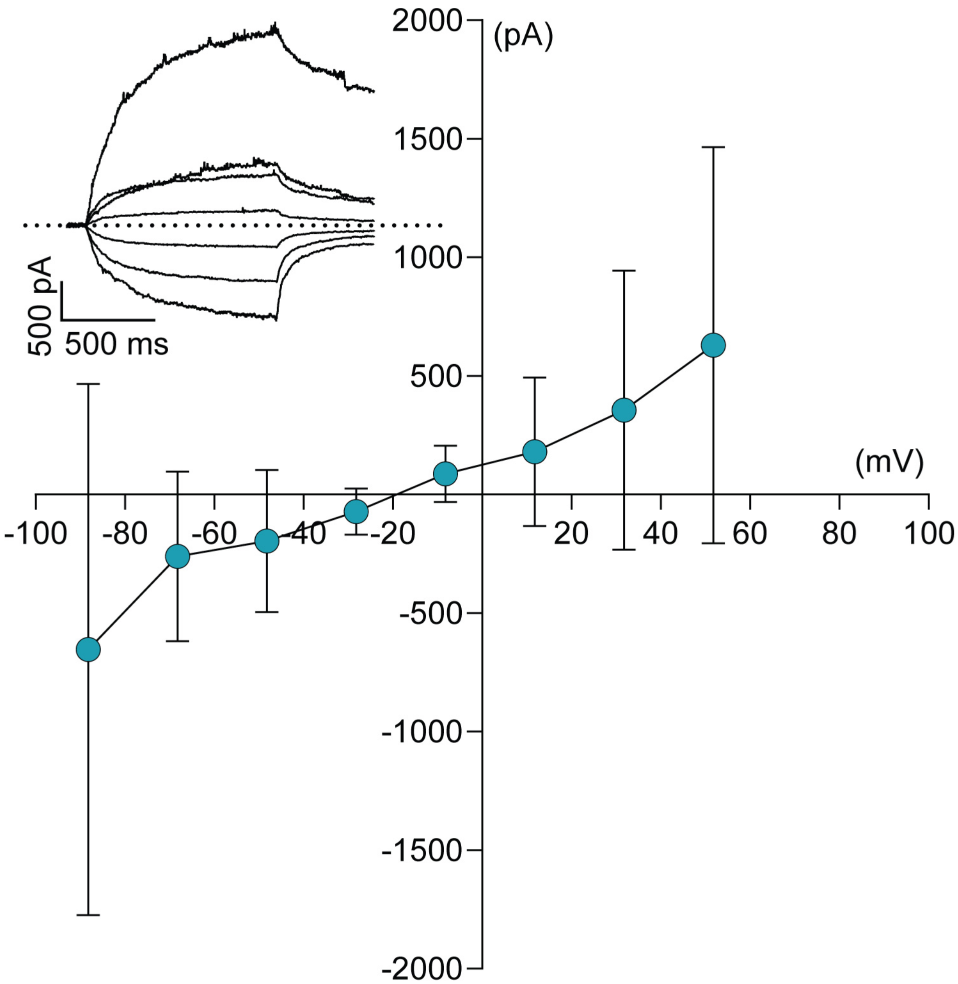

Supplementary Figure 8. Selectivity of FLYC1 K606E, K624E.
Average I–V curve of stretch-activated macroscopic currents from FLYC1 K606E, K624E (N=5 cells tested) in excised patches with 150 mM NaCl extracellular solution and 30 mM NaCl intracellular solution. Scatter plots are mean ± s.e.m. Inset, representative trace of macroscopic currents at membrane voltage (mV) of -88.2, -68.2, -48.2, -28.2, -8.2, 11.8, and 31.8.

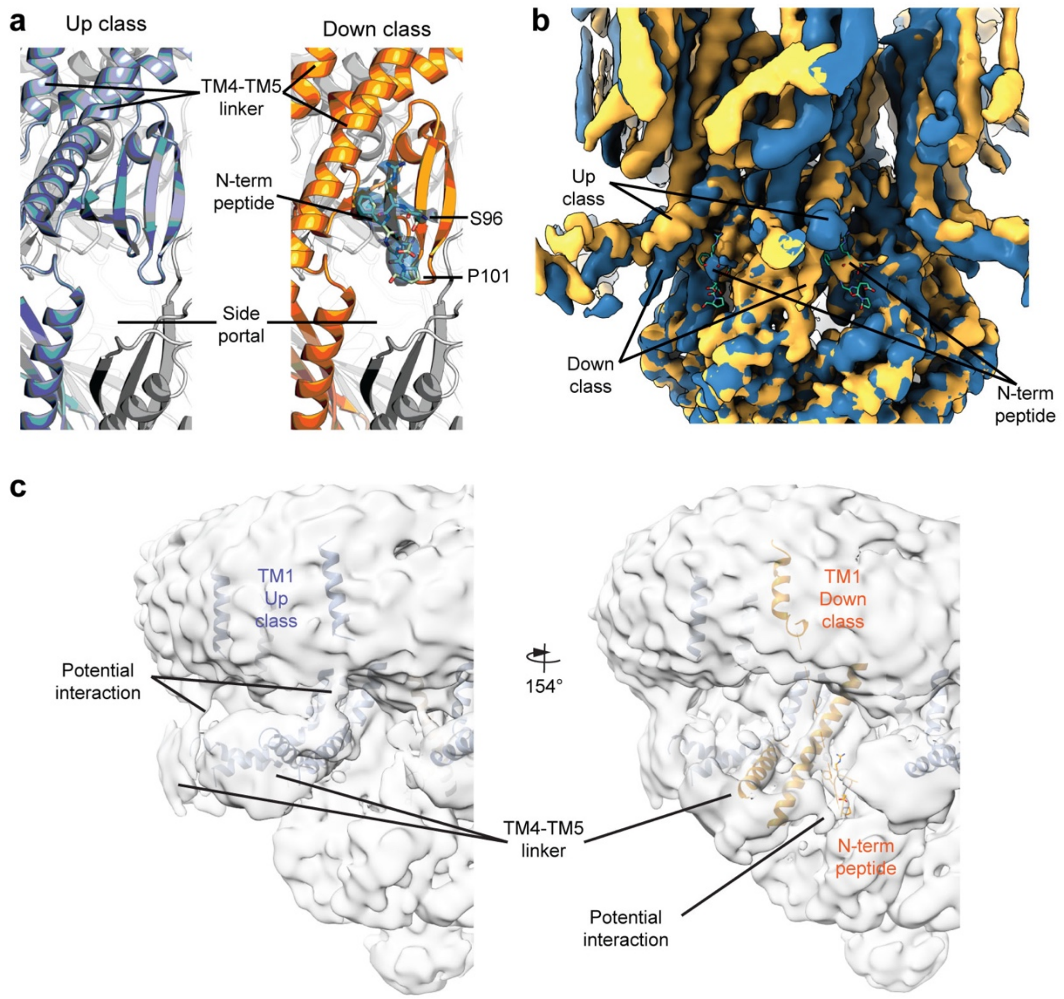

Supplementary Figure 9. Interaction of N-terminus of FLYC1.
a, A peptide density for part of the N-terminus is present in the down class. First and last residues built for the peptide are labeled. **b,** Superposed first (blue) and last frame (yellow) of variability analysis volume series showing the N-terminus peptide density limited to the down class. **c,** 6Å low-pass filter unsharpened C1 map showing density for potential interaction between TM4-TM5 linker and TM1, in the up class (left), or N-terminus peptide, in the down class (right).

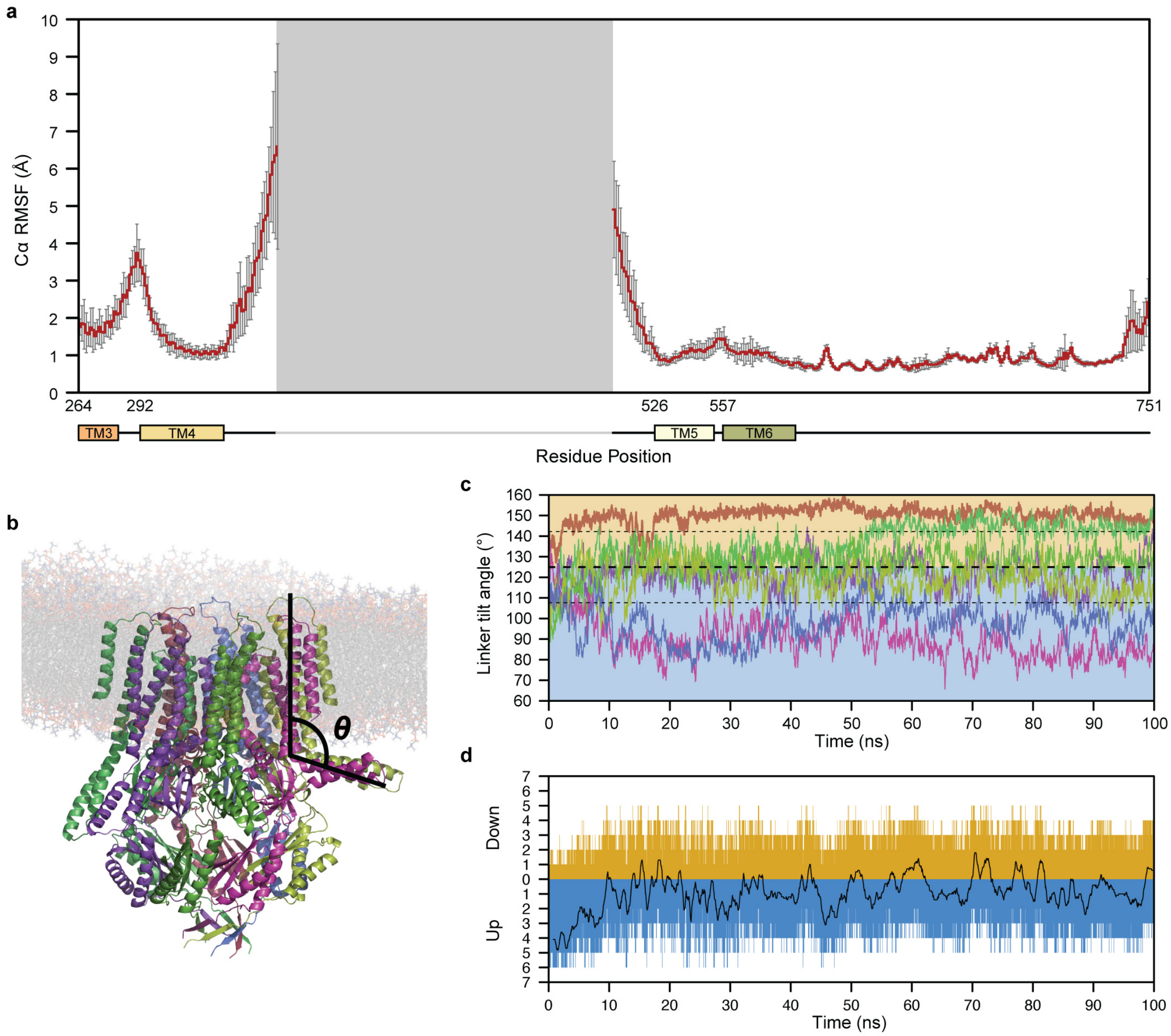

Supplementary Figure 10. Conformational flexibility of TM4-TM5 linker analyzed in an unrestrained simulation of the composite structure.
a, Root mean square fluctuation (RMSF) of Cα atoms. The red line represents an averaged RMSF value from all seven subunits with the error bars as ± 1 standard deviation. About 150 residues within the TM4-TM5 linker are unmodelled in our structure and are not present in the simulations (represented by the gray region). **b,** Snapshot at the end of the 100-ns simulation showing subunits initially in the "up" states now adopt a combination of "up" and "down" states. The linker tilt angle with respect to the *z*-axis is also illustrated. **c,** For the unrestrained simulation starting with the model with six of the seven subunits in the down states, the angle between each linker helix and the *z*-axis was plotted against simulation time. The average value between the starting angles (125º) is chosen as a criterion to classify individual subunits during the simulation to be in the "up" or the "down" state. **d,** The population of "up" and "down" states by frame (color bars) and as a rolling average across 20 frames (0.8 ns, black line).

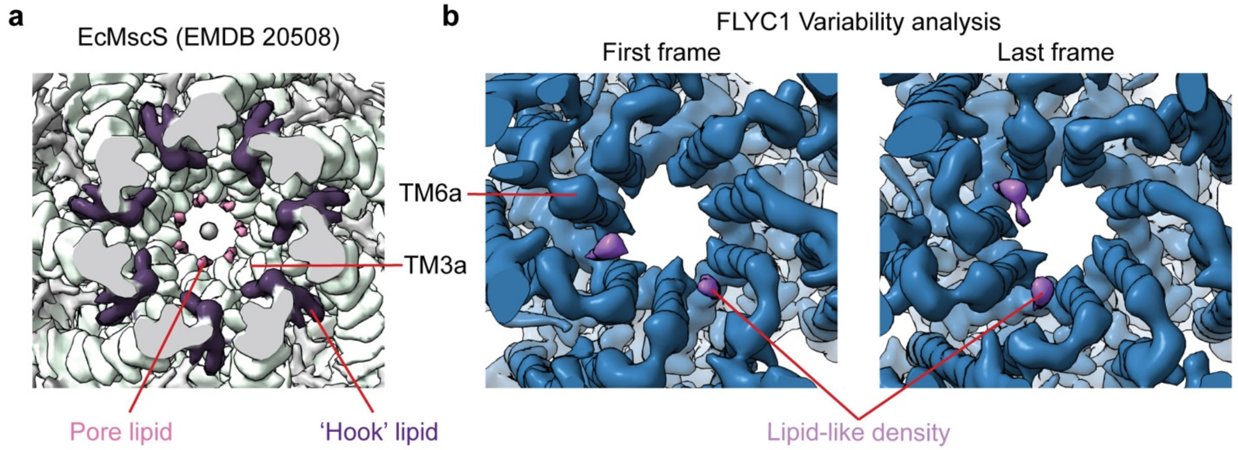

Supplementary Figure 11. FLYC1 and EcMscS lipid-like densities at the outer face of the pore.

a, Densities assigned to lipids in EcMscS map (EMDB 20508). Pore and 'hook' lipids are colored pink and dark purple, respectively. **b,** Maps corresponding to first (left) and last (right) frames of the first component from variability analysis of FLYC1. Lipid-like densities associated to protomers in the down conformation are colored in purple.

Supplementary Table 1. Data collection, processing, model refinement and validation.

	C1 map	Down focused map	Up focused map	Composite map
**Data collection and processing**				
Magnification	29000			
Voltage (kV)	300			
Electron exposure (e–/Å^2)	50			
Defocus range (μm)	-0.7 to -2.2			
Pixel size (Å)	1.03			
Initial particle images (no.)	959,656			
Symmetry imposed	C1	C1	C1	C1
Final particle images (no.)	129,933	145,362*	651,815*	
Map resolution (Å)	2.8	2.7	2.4	2.8†
FSC threshold	0.143	0.143	0.143	0.143
Map sharpening *B* factor (Å^2)	-47	-43	-38	
**Model**				
Composition				
Peptide chains	7	1‡	1‡	7
Protein residues	2176	386	371	2612
Ligands	7	1	1	7
R.m.s. deviations				
Bond lengths (Å)	0.023	0.022	0.022	0.022
Bond angles (°)	1.576	1.534	1.531	1.536
Validation				
MolProbity score	0.59	0.56	0.57	0.58
Clashscore	0.25	0.16	0.17	0.22
EMRinger score	4.07	3.32	3.64	4.14
Poor rotamers (%)	0.00	0.00	0.00	0.00
Ramachandran plot				
Favored (%)	98.84	98.66	99.17	98.94
Allowed (%)	1.16	1.34	0.83	1.06
Disallowed (%)	0.00	0.00	0.00	0.00
**Deposition ID**				
EMDB	24187	24188	24189	24186
PDB	7N5E	7N5F	7N5G	7N5D

*After symmetry expansion.
†Assigned resolution. Due to the final map being a composite map, resolution estimation based on FSC between half-maps could not be calculated.
‡Only protomer was used for model validation in the focused maps.

Supplementary Table 2. Counts of ion crossing events in simulations of the ‘all-up’ and ‘6 up 1 down’ composite models with an applied negative electric field. Each line represents an independent 100-ns simulation.

<table>
<tr><th>Model simulated</th><th>Electric field applied</th><th>Potential difference</th><th>Position restraints</th><th>Cl^- efflux events</th><th>Conductance G</th><th>Na^+ influx events (incomplete)</th></tr>
<tr><td rowspan="3">All up</td><td rowspan="3">–25 mV nm^{-1}</td><td rowspan="3">–425 mV</td><td rowspan="6">Backbone atoms</td><td>8</td><td>34 pS</td><td>1</td></tr>
<tr><td>17</td><td>71 pS</td><td>1</td></tr>
<tr><td>11</td><td>46 pS</td><td>1</td></tr>
<tr><td rowspan="4">6 up 1 down</td><td>–6.25 mV nm^{-1}</td><td>–112.5 mV</td><td>16</td><td>253 pS</td><td>2</td></tr>
<tr><td>–12.5 mV nm^{-1}</td><td>–225 mV</td><td>16</td><td>127 pS</td><td>10</td></tr>
<tr><td rowspan="2">–25 mV nm^{-1}</td><td rowspan="2">–450 mV</td><td>64</td><td>253 pS</td><td>12</td></tr>
<tr><td>None</td><td>29</td><td>115 pS</td><td>4</td></tr>
</table>

The number of chloride ions that traverse the channel end-to-end are counted i.e., those that enters the channel via the side portals and exit the channel through the ring of F572. While sodium ions enter the channel in the opposite direction, they remain in the cytoplasmic vestibule and never exit via the side portals in our 100-ns simulations and therefore were excluded in the estimation of conductance.
