# Supplementary material for: Structural insights into the Venus flytrap mechanosensitive ion channel Flycatcher1
Source: Nat Commun. 2022 Feb 14;13:850. doi: 10.1038/s41467-022-28511-5 (PMC8844309; doi:10.1038/s41467-022-28511-5)
Supplement: Supplementary file 3 — Description of Additional Supplementary Files [file 41467_2022_28511_MOESM3_ESM.pdf]

Description of Additional Supplementary Files

File name: Supplementary Movie 1

Description: Trajectory of a typical end-to-end ion crossing event in the presence of a potential difference of –450 mV. The movie covers a duration of 20 ns. Residues lining the side portal of entry are colored by type. The ring of F572 residues is colored in pink. Trajectory smoothing is applied (averaged across 8 ps).

File name: Supplementary Movie 2

Description: 3D EM Variability analysis of FLYC1. First variability component of FLYC1 after C7 symmetry expansion. Densities have been low-pass filtered to 6Å and high-pass filtered to 20Å. Appearance of N-terminus peptide and lipid-like density at the top of the channel are shown associated to the down class.
