# Supplementary material for: Structural insights into the Venus flytrap mechanosensitive ion channel Flycatcher1
Source: Nat Commun. 2022 Feb 14;13:850. doi: 10.1038/s41467-022-28511-5 (PMC8844309; doi:10.1038/s41467-022-28511-5)
Supplement: Supplementary file 6 — Reporting Summary [file 41467_2022_28511_MOESM6_ESM.pdf]

nature portfolio

Corresponding author(s): Ward, AB; Patapoutian, A; Murthy, SE

Last updated by author(s): Oct 21, 2021

Reporting Summary

Nature Portfolio wishes to improve the reproducibility of the work that we publish. This form provides structure for consistency and transparency in reporting. For further information on Nature Portfolio policies, see our Editorial Policies and the Editorial Policy Checklist.

Statistics

For all statistical analyses, confirm that the following items are present in the figure legend, table legend, main text, or Methods section.

n/a	Confirmed	
☐	☒	The exact sample size (*n*) for each experimental group/condition, given as a discrete number and unit of measurement
☐	☒	A statement on whether measurements were taken from distinct samples or whether the same sample was measured repeatedly
☐	☒	The statistical test(s) used AND whether they are one- or two-sided<br>*Only common tests should be described solely by name; describe more complex techniques in the Methods section.*
☒	☐	A description of all covariates tested
☒	☐	A description of any assumptions or corrections, such as tests of normality and adjustment for multiple comparisons
☐	☒	A full description of the statistical parameters including central tendency (e.g. means) or other basic estimates (e.g. regression coefficient) AND variation (e.g. standard deviation) or associated estimates of uncertainty (e.g. confidence intervals)
☐	☒	For null hypothesis testing, the test statistic (e.g. *F*, *t*, *r*) with confidence intervals, effect sizes, degrees of freedom and *P* value noted<br>*Give P values as exact values whenever suitable.*
☒	☐	For Bayesian analysis, information on the choice of priors and Markov chain Monte Carlo settings
☒	☐	For hierarchical and complex designs, identification of the appropriate level for tests and full reporting of outcomes
☒	☐	Estimates of effect sizes (e.g. Cohen's *d*, Pearson's *r*), indicating how they were calculated

Our web collection on statistics for biologists contains articles on many of the points above.

Software and code

Policy information about availability of computer code

Data collection	Electrophysiology data was collected using Clampex v10.6 from Molecular Devices. Leginon software was used for automated collection of EM data (Suloway et al., 2005).
Data analysis	For electrophysiology data analysis, we used Clampfit v10.6, GraphPad Prism v9, and Microsoft Excel. Micrograph movie frames were aligned and dose-weighted using MotionCor2(Zheng et al., 2017). Initial data processing was performed in cryoSPARCv2.15 (Punjani et al., 2017). GCTF (Zhang, 2016) was used for estimation of CTF parameters. Particle stacks were transferred to Relion/3.0 (Zivanov et al., 2018) for further processing.

For manuscripts utilizing custom algorithms or software that are central to the research but not yet described in published literature, software must be made available to editors and reviewers. We strongly encourage code deposition in a community repository (e.g. GitHub). See the Nature Portfolio guidelines for submitting code & software for further information.

Data

Policy information about availability of data

All manuscripts must include a data availability statement. This statement should provide the following information, where applicable:

- Accession codes, unique identifiers, or web links for publicly available datasets
- A description of any restrictions on data availability
- For clinical datasets or third party data, please ensure that the statement adheres to our policy

3D maps and models from the EM analysis have been deposited to the Electron Microscopy Databank (http://www.emdatabank.org/) and the Protein Data Bank (http://www.rcsb.org/), respectively. The accession numbers are listed in the table included in the manuscript.

Field-specific reporting

Please select the one below that is the best fit for your research. If you are not sure, read the appropriate sections before making your selection.

☒ Life sciences ☐ Behavioural & social sciences ☐ Ecological, evolutionary & environmental sciences

For a reference copy of the document with all sections, see nature.com/documents/nr-reporting-summary-flat.pdf

Life sciences study design

All studies must disclose on these points even when the disclosure is negative.

Sample size	For electrophysiology, sample sizes represent the number of cells used for recordings and analysis. The sizes were chosen based on consistency and quality of data across conditions and multiple experiments.
Data exclusions	For patch clamp recordings, no data was excluded unless the recording quality was poor due to factors such as large noise or patch instability.
Replication	Electrophysiological experiments were reproduced according to the sample size as indicated in each figure. In addition, the recordings and parameters were reproducible across multiple days and transfection batches.
Randomization	For patch clamp recordings, samples were grouped based on the genes of interests transfected into the cells.
Blinding	The investigators were not blinded to group allocation.

Reporting for specific materials, systems and methods

We require information from authors about some types of materials, experimental systems and methods used in many studies. Here, indicate whether each material, system or method listed is relevant to your study. If you are not sure if a list item applies to your research, read the appropriate section before selecting a response.

Materials & experimental systems

n/a	Involved in the study	
☒	☐	Antibodies
☐	☒	Eukaryotic cell lines
☒	☐	Palaeontology and archaeology
☒	☐	Animals and other organisms
☒	☐	Human research participants
☒	☐	Clinical data
☒	☐	Dual use research of concern

Methods

n/a	Involved in the study	
☒	☐	ChIP-seq
☒	☐	Flow cytometry
☒	☐	MRI-based neuroimaging

Eukaryotic cell lines

Policy information about cell lines

Cell line source(s)	HEK293T Piezo1 knockout cells were created in house using CRISPR-Cas9 system. The methods for generating the cell line has been published, and is described in detail in Lukacs 2015 (PMID: 26387913). HEK293F and HEK293T cell lines were used for protein production.
Authentication	The HEK293T Piezo1 Knockout cells were validated by genotyping, as mentioned in Lukacs 2015.
Mycoplasma contamination	Cells were tested for mycoplasma contamination and reported negative.
Commonly misidentified lines (See ICLAC register)	*Name any commonly misidentified cell lines used in the study and provide a rationale for their use.*
