# Supplementary figures and images for: Structural insights into the Venus flytrap mechanosensitive ion channel Flycatcher1
Source: Nat Commun. 2022 Feb 14;13:850. doi: 10.1038/s41467-022-28511-5 (PMC8844309; doi:10.1038/s41467-022-28511-5)

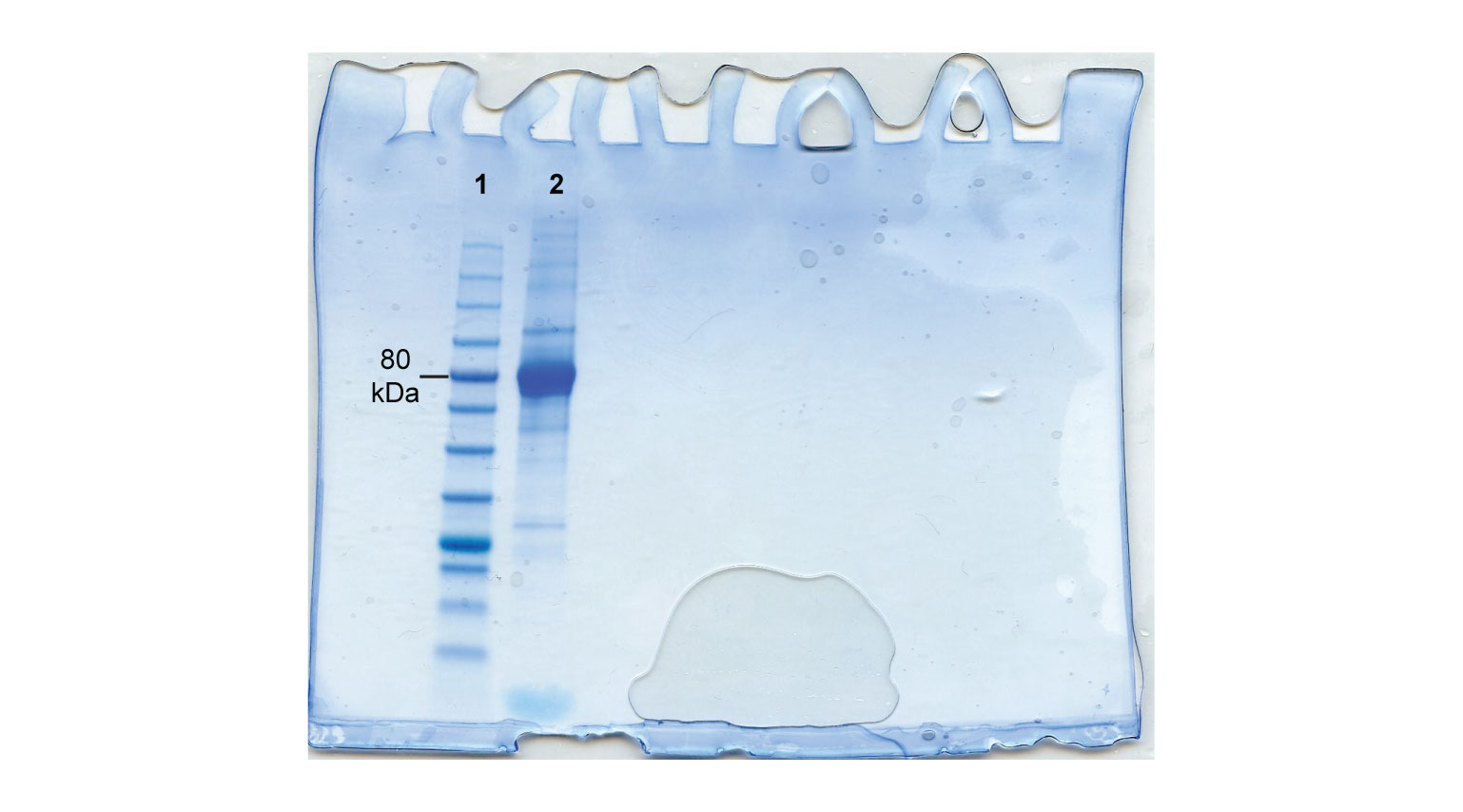

Supplement: Supplementary file 7 — Source Data [file 41467_2022_28511_MOESM7_ESM.zip › Source Data 1.jpg]
